# CCPG1, a cargo receptor required for reticulophagy and endoplasmic reticulum proteostasis

**DOI:** 10.1080/15548627.2018.1441473

**Published:** 2018-06-19

**Authors:** Matthew D. Smith, Simon Wilkinson

**Affiliations:** Edinburgh Cancer Research UK Centre, MRC Institute of Genetics and Molecular Medicine, University of Edinburgh, Edinburgh, UK

**Keywords:** cargo receptors, exocrine pancreas, FIP200, inflammation, proteostasis, rough endoplasmic reticulum, unfolded protein response

## Abstract

The importance of selective macroautophagy/autophagy in cellular health is increasingly evident. The selective degradation of portions of the endoplasmic reticulum (ER), or reticulophagy, is an emerging example but requires further mechanistic detail and broad evidence of physiological relevance. In a recent study, we identified CCPG1, an ER-resident transmembrane protein that can bind to Atg8-family proteins and, independently and discretely, to RB1CC1/FIP200. Both of these interactions are required to facilitate CCPG1's function as a reticulophagy cargo receptor. *CCPG1* transcripts are inducible by ER stress, providing a direct link between ER stress and reticulophagy. In vivo, CCPG1 prevents the hyper-accumulation of insoluble protein within the ER lumen of pancreatic acinar cells and alleviates ER stress. Accordingly, CCPG1 loss sensitizes the exocrine pancreas to tissue injury.

The endoplasmic reticulum is a key intracellular organelle that oversees the synthesis and quality control of transmembrane and secretory proteins. If environmental conditions become unfavorable or the ER becomes overwhelmed, proteins can become misfolded and are retained in the ER lumen. This can be defined as a form of ER stress. The best understood mechanism for resolving ER stress is the unfolded protein response (UPR). The UPR employs sensors that detect unfolded protein levels in the ER lumen and activate downstream signalling cascades in the cytosol and nucleus. This leads to inhibition of global translation and transcriptional upregulation of a subset of genes that act to restore ER homeostasis.

Whereas it is known that the UPR drives increased global autophagic flux through the transcriptional upregulation of *ATG* genes, there is no evidence for this having a specific effect on the ER. In recent years, our understanding of the selective degradation of the mammalian ER, known as reticulophagy, has grown with the discovery of a number of reticulophagy receptors, namely RETREG1/FAM134B, SEC62 and RTN3. Despite this, no direct link has been proposed between ER stress, the UPR and the induction of reticulophagy.

We recently identified CCPG1 in an affinity purification-mass spectrometry screen for GABARAP-interacting proteins. CCPG1 is an ER-resident, vertebrate-specific, single-pass transmembrane protein that possesses both cytosolic and ER-lumenal regions (Figure 1). CCPG1 traffics to phagophores (the precursors to autophagosomes) upon amino acid starvation. As with other autophagy cargo receptors, CCPG1 interacts with Atg8-family proteins through a canonical LIR motif located in the cytosolic region. This LIR motif is required for degradation by autophagy and for function as a reticulophagy receptor. Interestingly, a secondary affinity purification-mass spectrometry screen uncovered a direct interaction of CCPG1 with RB1CC1. RB1CC1 binding was also mapped to the cytosolic portion of CCPG1 protein at 2 sites not involved in Atg8-family binding. We termed these polypeptide sequences RB1CC1/FIP200-interacting region (FIR) motifs. Binding to RB1CC1 is required for CCPG1 trafficking and promotion of reticulophagy. This begs the question: Why does CCPG1 require RB1CC1 binding whereas Atg8-family binding alone is sufficient in other cargo receptors? In yeast, many selective autophagy receptors (SARs) bind to *both* Atg11 and Atg8. In the case of CCPG1, the dominant FIR has sequence homology to the Atg11-binding consensus sequence of yeast SARs. Additionally, CCPG1 binds to a C-terminal portion of RB1CC1 that encompasses its Atg11-homology region. These observations raise the possibility that CCPG1 employs an analogous mechanism to that of yeast SARs. In yeast, the interaction between SARs and Atg11 allows recruitment of active Atg1 to the cargo. This should be tested for CCPG1 and ULK1 recruitment.

**Figure 1.**
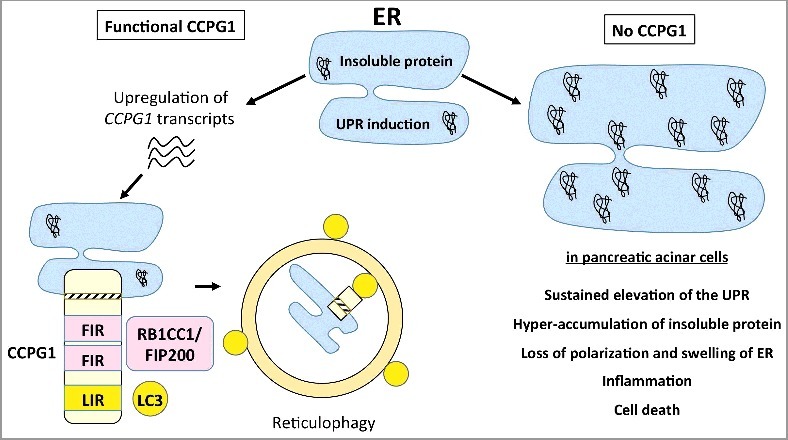
Model of CCPG1 function in ER proteostasis. The accumulation of insoluble protein within the ER lumen causes induction of the UPR. When CCPG1 is present, transcripts of *CCPG1* are upregulated via the UPR. This results in an increase in CCPG1 protein at the ER. CCPG1 possesses FIR and LIR motifs, allowing interaction with the autophagic proteins RB1CC1/FIP200 and those of the Atg8-family, respectively. These interactions facilitate the sequestration of portions of the ER into phagophores. This process restores ER homeostasis. When CCPG1 is not present in the pancreatic acinar cell in vivo, insoluble proteins become hyper-accumulated, leading to ER disruption and sustained, high levels of the UPR. In older mice, this phenomenon co-presents with the presumed sequelae of acinar cell death and sensitivity to pancreatic inflammation.

The physiological importance of reticulophagy requires further elucidation. Most evidence for ER disruption following autophagy loss comes from models where all autophagy has been removed. As such, interpretation of the effects on ER that are observed is often confounded by other, clearly primary, effects, such as loss of mitophagy and the subsequent bioenergetic compromise of the cell. A more direct study showed that *Retreg1*/*Fam134b* mutant mice display a loss of maintenance of cellular health of peripheral sensory neurones, which correlates with distended ER in these cells. However, a role for reticulophagy in most organs remains unaddressed. Thusly, *Ccpg1* hypomorphic mice were generated. Prior to this, we had also shown that *CCPG1* transcripts were inducible by ER stress, in human established cell lines in vitro. Thus, in vivo, we decided to look at the acinar cells of the exocrine pancreas, which, due to their high biosynthetic workload, are under constitutive low-level ER stress. *Ccpg1*-deficient mice exhibit hyper-accumulation of insoluble protein within the ER lumen of these cells. This is associated with elevated, sustained levels of ER stress, tissue injury and an inflammatory response in the pancreas (Figure 1). We therefore propose that CCPG1 maintains ER lumenal proteostasis in the exocrine pancreas, maintaining ER stress at sustainable levels. Of course, additional functions of CCPG1 in ER homeostasis that are independent of reticulophagy cannot be ruled out and should be addressed in future studies.

In summary, CCPG1 constitutes an example of a noncanonical reticulophagy cargo receptor, due to its functional requirement to bind Atg8-family proteins as well as RB1CC1, independently of one another. The *CCPG1* gene is inducible by the UPR, providing insight into how cells are able to respond to ER stress through reticulophagy. In vivo, CCPG1 is required to maintain the health of pancreatic acinar cells and, as such, future work in disease-state scenarios, such as pancreatic cancer formation, which is regulated by autophagy status, will further illuminate the role of CCPG1 and reticulophagy in health and disease.

